# Characteristics of the chloroplast genome and genetic divergence of *Tamarix hispida* Willd. 1816 (Tamaricaceae)

**DOI:** 10.1080/23802359.2024.2383686

**Published:** 2024-07-26

**Authors:** Haowen Tian, Xiaojun Shi, Hongxiang Zhang

**Affiliations:** a College of Life Sciences, Xinjiang Key Laboratory for Ecological Adaptation and Evolution of Extreme Environment Biology, Xinjiang Agricultural University, Urumqi, China; b State Key Laboratory of Desert and Oasis Ecology, Key Laboratory of Ecological Safety and Sustainable Development in Arid Lands, Xinjiang Institute of Ecology and Geography, Chinese Academy of Sciences, Urumqi, China; c Xinjiang Key Laboratory of Conservation and Utilization of Gene Resources, Urumqi, China; d Specimen Museum of Xinjiang Institute of Ecology and Geography, Chinese Academy of Sciences, Urumqi, China

**Keywords:** *Tamarix hispida*, chloroplast genome, phylogenetic analysis, divergence time

## Abstract

*Tamarix hispida* Willd. 1816, a crucial native plant species in the arid desert region of northwestern China, plays a significant role in maintaining ecological stability. It is instrumental in addressing soil salinity–alkalinity and heavy metal pollution. This research aims to analyze the phylogenetic divergence pattern and evolutionary history of *T. hispida* by comparing chloroplast genome structures across different populations. Despite the minimal differences in chloroplast genome structure due to conserved genes and junction regions, sequencing was conducted using the Illumina NovaSeq platform to verify the historical evolutionary processes between different populations, followed by assembly and annotation. The results revealed that the *T. hispida* chloroplast genome is approximately 156,164–156,186 bp in length, with a quadripartite structure and 131 annotated genes. Phylogenetic analysis indicated two lineages within *T. hispida*, with a divergence time of 3.15 Ma. These findings emphasize the low genetic diversity in *T. hispida* and offer valuable insights into its evolutionary past. To effectively protect and manage this species, increased scientific research and monitoring of its genetic diversity are necessary. This study underscores the importance of comprehending the genetic mechanisms behind species divergence to develop informed conservation strategies.

## 1. Introduction

The *Tamarix hispida* Willd. 1816, a significant native plant species in the arid desert region of northwestern China within the *Tamarix* genus, plays a vital role in maintaining ecological stability (Gaskin 2003). It aids in the restoration of soil salinity–alkalinity and heavy metal pollution (Pang et al. 2022; Xie et al. 2023). Nevertheless, self-pollination tendencies and occasional secondary flowering in the *Tamarix* genus have caused significant hybridization, complicating the accurate identification of *Tamarix* species (Terrones and Juan 2023). The *Tamarix* genus, belonging to the family Tamaricaceae, comprises approximately 70–75 recognized species, many of which are adapted to extreme environmental conditions (Villar et al. 2019). Chloroplast genome sequences unravel extensive sequence and structural diversities within and among plant species, offering valuable insights into comprehending climate adaptation in economically crucial crops, facilitating the breeding of closely related species, and identifying and conserving valuable traits (Llorente et al. 2021). Despite the significant role of chloroplast genomes in phylogenetic studies, there is a lack of comprehensive phylogenetic research on *Tamarix* species, making it essential to explore their genetic diversity and evolutionary history.

**Figure 1.**
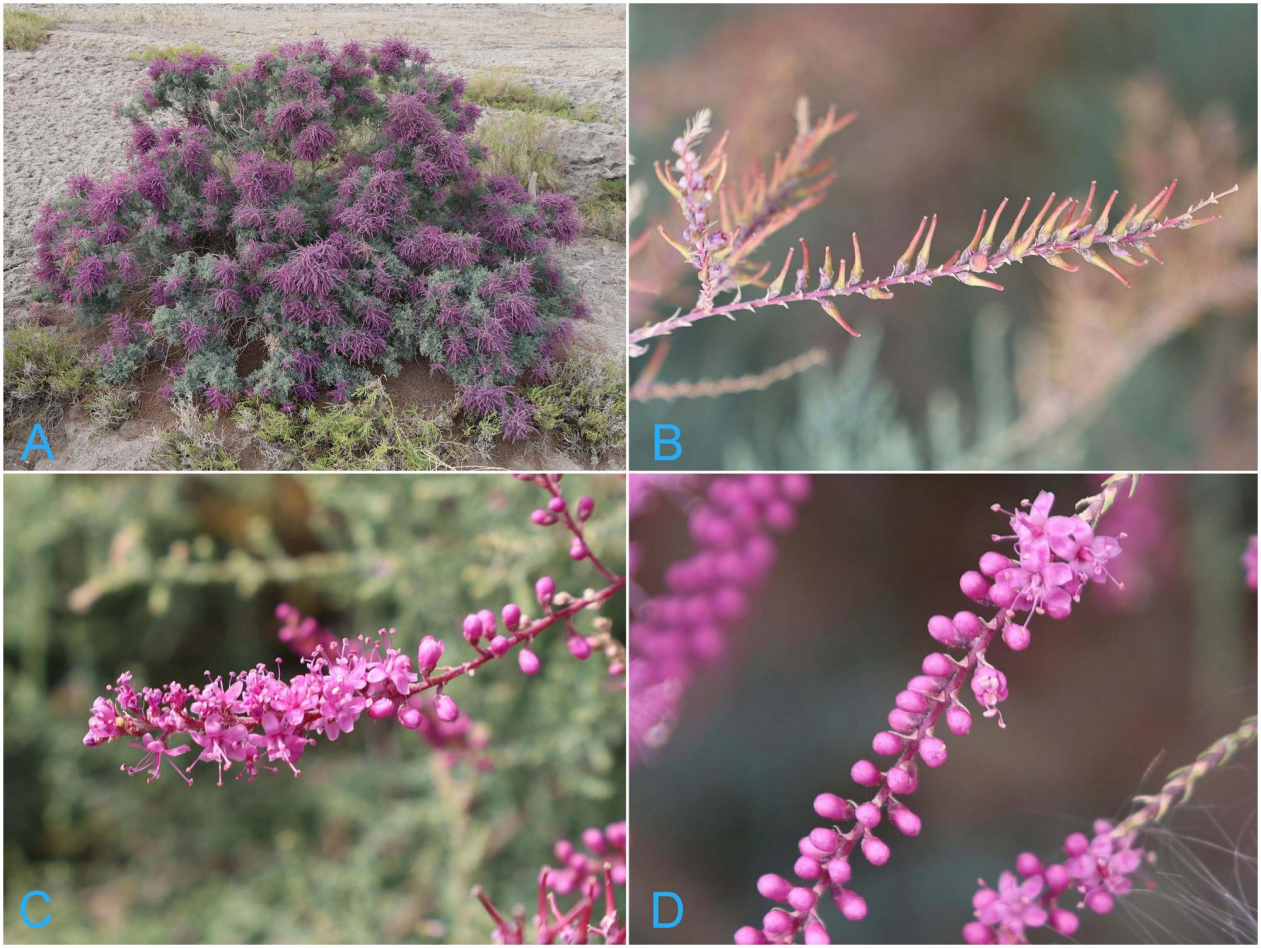
*Tamarix hispida*: vegetative morphology. (A) Plant; (B) capsule (conic); (C) petals (petals 5, spreading, reflexed in upper part, purple-red or red); (D) inflorescence. Photo taken by Haowen Tian in Yopurga, Xinjiang.

To enhance the differentiation of *T. hispida* and investigate potential discrepancies in chloroplast genomes among distinct lineages, we conducted a comprehensive study to establish a benchmark for future chloroplast genome research on other species within the Tamaricaceae family. Given the highly preserved nature of chloroplast genes, our sampling encompassed individuals from various locations, comprising a total of nine *T. hispida* specimens. Each specimen was deliberately selected at a minimum distance of 100 km from one another to evaluate the possible impact of geographic isolation on chloroplast genomes. Genetic diversity in chloroplast genomes reflects the historical evolution and geographical dispersion of plants, along with the phylogenetic associations among various species (Figure 1).

## 2. Materials and methods

### 2.1. Experimental materials, sequencing, and chloroplast genome assembly

Leaf samples were collected from nine populations of *T. hispida* in Xinjiang, China, covering the primary distribution area of the species (Table 1). Voucher specimens were stored at the Specimen Museum of Xinjiang Institute of Ecology and Geography, Chinese Academy of Sciences (XJBI, Hongxiang Zhang, zhanghx561@ms.xjb.ac.cn) (Table S1). The samples underwent DNA extraction at Shanghai Personalbio Technology Co., Ltd. (Shanghai, China) using the improved CTAB method (Porebski et al. 1997). Subsequently, we evaluated the DNA extraction quality via 0.8% agarose gel electrophoresis and quantified the DNA with a UV spectrophotometer. A library comprising 400 bp insert fragments was then constructed on the Illumina NovaSeq platform (San Diego, CA), and paired-end sequencing was performed to acquire 150 bp sequences from both ends of each read. The sequencing depth is shown in Figure S1. Raw data quality control was conducted using Fastp v0.23.1 (Chen et al. 2018), and assembly was performed with GetOrganelle v 1.7.5 (Jin et al. 2020) software. The chloroplast genome was annotated using PGA (Qu et al. 2019) software and assembly results were manually refined with Geneious v 9.0.2 (Kearse et al. 2012) software. The assembled chloroplast genome was deposited in the NCBI database (Table S1). OGDRAW was utilized to create a circular map of the chloroplast (https://chlorobox.mpimp-golm.mpg.de/OGDraw.html). CPGview (Liu et al. 2023) was used to generate cis-spliced and trans-spliced gene illustrations, as shown in Figures S2 and S3.

**Figure 2.**
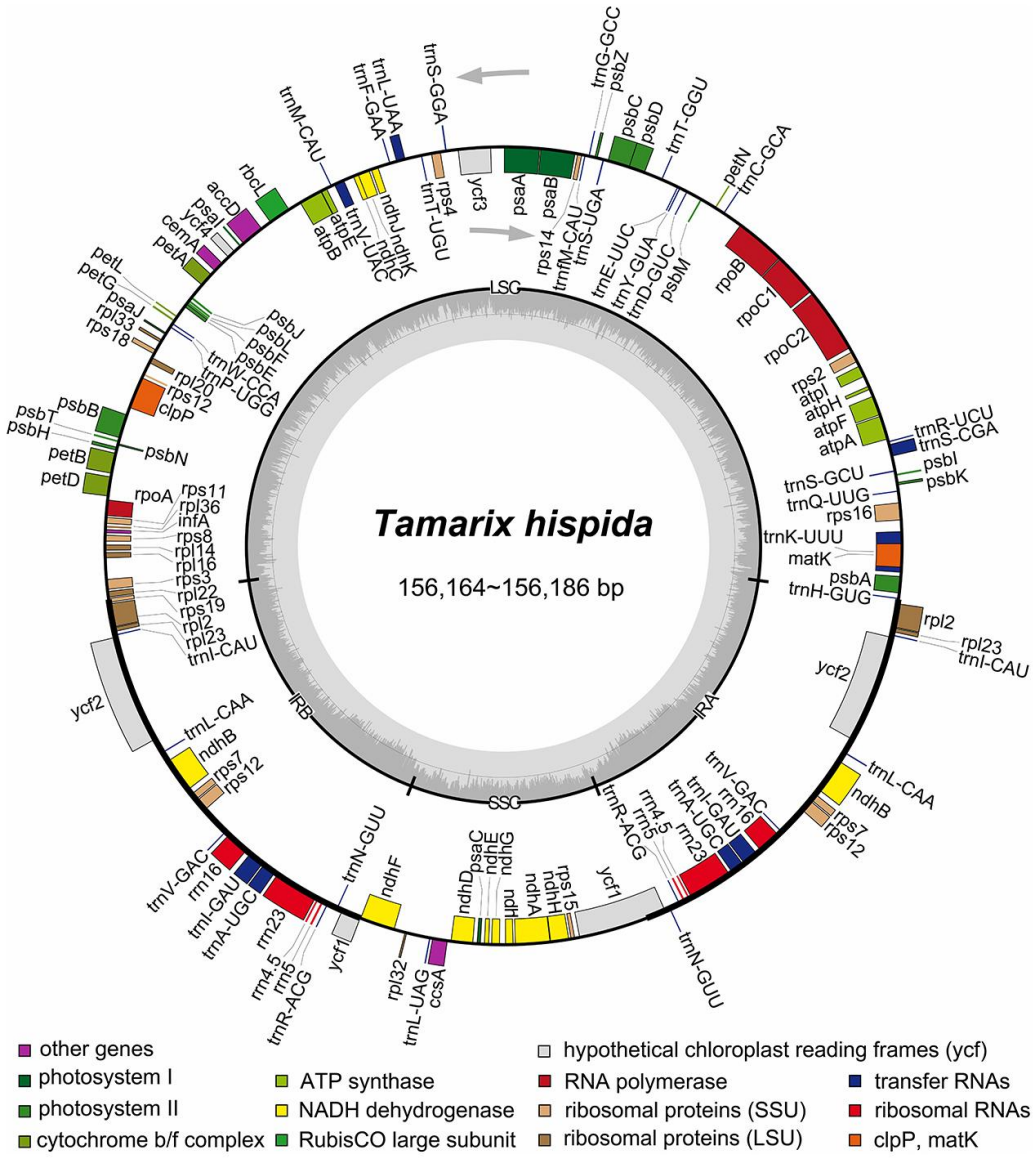
Gene maps of *Tamarix hispida* chloroplast genomes. Genes located on the inner side of the circle undergo transcription in a clockwise direction, whereas genes on the outer side are transcribed in a counterclockwise direction. Dark gray and light gray color represent guanine and cytosine (GC) content and adenine and thymine (AT) content, respectively.

**Figure 3.**
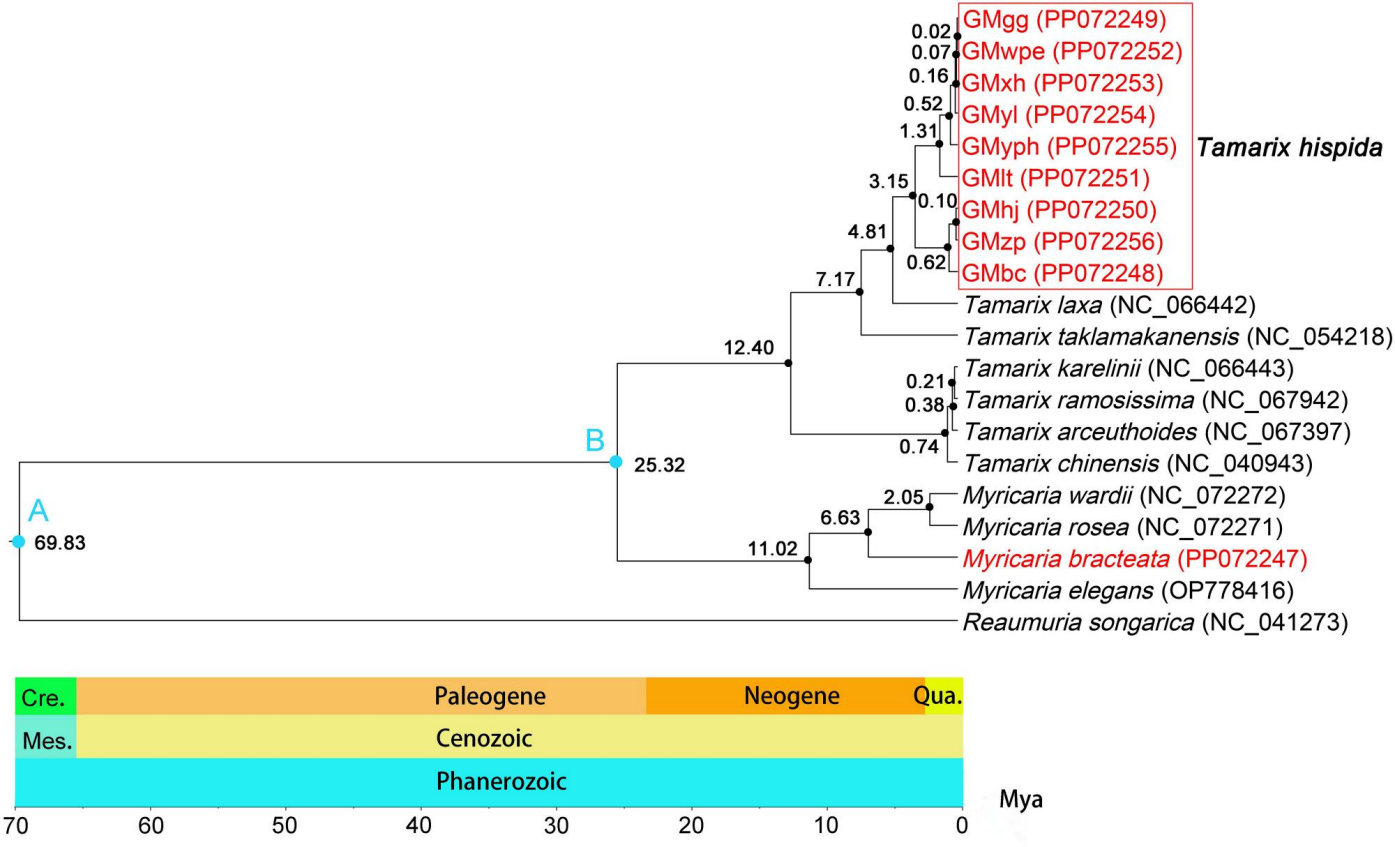
The phylogenetic tree and divergence time of the genus Tamaricaceae were constructed using BEAST software based on chloroplast genome sequences. Fossil calibration points A and B were used to calibrate the tree. Geological periods are shown at the bottom. Cre., Mes., and Qua. represent Cretaceous, Mesozoic, and Quaternary periods, respectively. The analysis reveals two main lineages of *T. hispida*, diverging approximately 3.15 million years ago. The following sequences were used: *Reaumuria songarica* (accession number: NC_041273, Yao et al. 2019), *Myricaria elegans* (accession number: OP778416, unpublished), *Myricaria bracteata* (accession number: PP072247, this study), *Myricaria rosea* (accession number: NC_072271, unpublished), *Myricaria wardii* (accession number: NC_072272, unpublished), *Tamarix chinensis* (accession number: NC_040943, Yao et al. 2019), *Tamarix arceuthoides* (accession number: NC_067397, unpublished), *Tamarix ramosissima* (accession number: NC_067942, unpublished), *Tamarix karelinii* (accession number: NC_066443, unpublished), *Tamarix taklamakanensis* (accession number: NC_054218, unpublished), *Tamarix laxa* (accession number: NC_066442, unpublished), GMbc (accession number: PP072248, this study), GMzp (accession number: PP072256, this study), GMhj (accession number: PP072250, this study), GMlt (accession number: PP072251, this study), GMyph (accession number: PP072255, this study), GMyl (accession number: PP072254, this study), GMxh (accession number: PP072253, this study), GMwpe (accession number: PP072252, this study), and GMgg (accession number: PP072249, this study).

**Table 1.** Sampling locations of nine *Tamarix hispida* populations.

Population code	Locality	Longitude	Latitude
GMbc	Bachu, Xinjiang	78.5028	39.7985
GMgg	Tuokexun, Xinjiang	88.5531	42.5945
GMhj	Hejing, Xinjiang	86.4750	42.2106
GMIt	Bugur, Xinjiang	84.7892	41.6112
GMwpe	Shufu, Xinjiang	75.5468	39.2530
GMxh	Xinhe, Xinjiang	82.5736	41.6587
GMyl	Yuli, Xinjiang	86.4083	41.1596
GMyph	Yopurga, Xinjiang	76.4980	39.2727
GMzp	Poskam, Xinjiang	77.2671	38.2362

### 2.2. Phylogenetic analysis

To elucidate the phylogenetic relationship among nine populations of *T. hispida* and other *Tamarix* species, chloroplast genomes of 10 Tamaricaceae species (Table S1) were also downloaded from the GenBank database, with *Reaumuria songarica* (NC_041273) as an outgroup. In total, nine *T. hispida* samples, six other *Tamarix* species, one *Reaumuria* specie, and four *Myricaria* species were used in the phylogenetic tree construction. The MAFFT v7.520 software was subsequently utilized for alignment, and the resulting FASTA file was converted into a NEX file format using Geneious software. The BEAST v1.6.1 software was then employed to estimate divergence time, with two fossil calibration points at 25 Ma and 70 Ma (Zhang et al. 2014). The chain length of MCMC was set to 1,000,000,000, while all other parameters were retained with default values. Finally, the ML phylogenetic tree (Wu et al. 2015) was constructed using IQ-TREE v2.2.2.6 (Nguyen et al. 2015), which selected the best model as TVM + F + I + G4.

## 3. Results

### 3.1. Basic characteristics and comparative analysis of the chloroplast genomes

The complete chloroplast genome of *T. hispida* (accession numbers: PP072247–PP072256) spans a length of 156,164–156,186 bp, encompassing a large single-copy (LSC, 84,791–84,807 bp) region, a small single-copy (SSC, 18,250–18,257 bp) region, and two inverted repeats (IRs, 26,561 bp each) (Figure 2). The GC contents of the LSC, SSC, and IR regions are 34.16%, 29.58–29.61%, and 42.51%, respectively, culminating in an overall GC content of 36.5% for the *T. hispida* chloroplast genome. The genome possesses 131 genes, comprising 85 protein-coding genes, 37 tRNA genes, and eight rRNA genes. Each of thirteen protein-coding genes (*rpo*C1, *atp*F, *rps*16, *pet*B, *pet*D, two *rpl*2, *rpl*16, *ndh*A, and two *ndh*B) and seven tRNA genes (two *trn*A-UGC, two *trn*I-GAU, *trn*K-UUU, *trn*L-UAA, *trn*S-CGA, and *trn*V-UAC) contain one intron, while three protein-coding genes (two *rps*12, *ycf*3, and *clp*P) contain two introns.

### 3.2. Phylogenetic relationships

The maximum-likelihood (ML) tree obtained from IQ-TREE correlated with the divergence time tree generated by BEAST; hence, the ML tree result was omitted from the phylogenetic analysis (Figure 3). *T. hispida* owns the closest relationship with *T. laxa*, and a more distant relationship with *Reaumuria* than *Myricaria*, in consistent with the prevailing morphological classification. The nine *T. hispida* populations were clustered into two lineages. The divergence between *T. hispida* and related species occurred at 4.81 Ma, whereas the divergence between lineage one and lineage two took place at 3.15 Ma.

## 4. Discussion and conclusions

The genus *Tamarix* originated in the ancient Mediterranean coastal region, with its present-day distribution influenced by factors like the retreat of the Tethys Sea, tectonic movements since the Tertiary period, and the impacts of Quaternary glaciation and interglacial cycles (Daoyuan et al. 2003; Anzidei et al. 2014). *Tamarix* tends to be easy hybridization. Due to varying degrees of gene flow, species with different morphological characteristics coexist in the same area (Sheidai and Koohdar 2023). Hence, hybridization may contribute to the divergence of the two lineages. Explaining the variation resulting from hybridization at the chloroplast genome level proves to be a challenging task. Therefore, further research in diverse areas is needed to provide a more comprehensive and clear understanding. Importantly, the constructed phylogenetic tree in this study indicates that the complete chloroplast genome can serve as a valuable tool for species identification.

## Supplementary Material

Supplemental Material.docx

## Data Availability

The data that support the findings of this study are openly available in GenBank of NCBI at https://www.ncbi.nlm.nih.gov, reference number GMbc (PP072248), GMgg (PP072249), GMhj (PP072250), GMlt (PP072251), GMwpe (PP072252), GMxh (PP072253), GMyl (PP072254), GMyph (PP072255), and GMzp (PP072256). The *T. hispida* associated BioProject, SRA, and Bio-Sample numbers are PRJNA1083937, SRR28226810–SRR28226818, and SAMN40262530–SAMN40262538, respectively.
