# Supplementary material for: Characteristics of the chloroplast genome and genetic divergence of Tamarix hispida Willd. 1816 (Tamaricaceae)
Source: Mitochondrial DNA B Resour. 2024 Jul 26;9(7):915–9. doi: 10.1080/23802359.2024.2383686 (PMC11285246; doi:10.1080/23802359.2024.2383686)
Supplement: Supplemental Material.docx [file TMDN_A_2383686_SM4545.docx]

Table S1. GenBank number used for constructing the phylogenetic tree

| Genbank | species | Voucher specimens |
| --- | --- | --- |
| NC_040943 | *Tamarix chinensis* |  |
| NC_054218 | *Tamarix taklamakanensis* |  |
| NC_066442 | *Tamarix laxa* |  |
| NC_066443 | *Tamarix karelinii* |  |
| NC_067397 | *Tamarix arceuthoides* |  |
| NC_067942 | *Tamarix ramosissima* |  |
| NC_072271 | *Myricaria rosea* |  |
| NC_072272 | *Myricaria wardii* |  |
| NC_041273 | *Reaumuria songarica* |  |
| OP778416 | *Myricaria elegans* |  |
| PP072247 | *Myricaria bracteata* | 2023WQ1 |
| PP072248 | GMbc | 2023BC1 |
| PP072249 | GMgg | 2023GG1 |
| PP072250 | GMhj | 2023HJ1 |
| PP072251 | GMlt | 2023LT1 |
| PP072252 | GMwpe | 2023WPE1 |
| PP072253 | GMxh | 2023XH1 |
| PP072254 | GMyl | 2023YL1 |
| PP072255 | GMyph | 2023YPH1 |
| PP072256 | GMzp | 2023ZP1 |


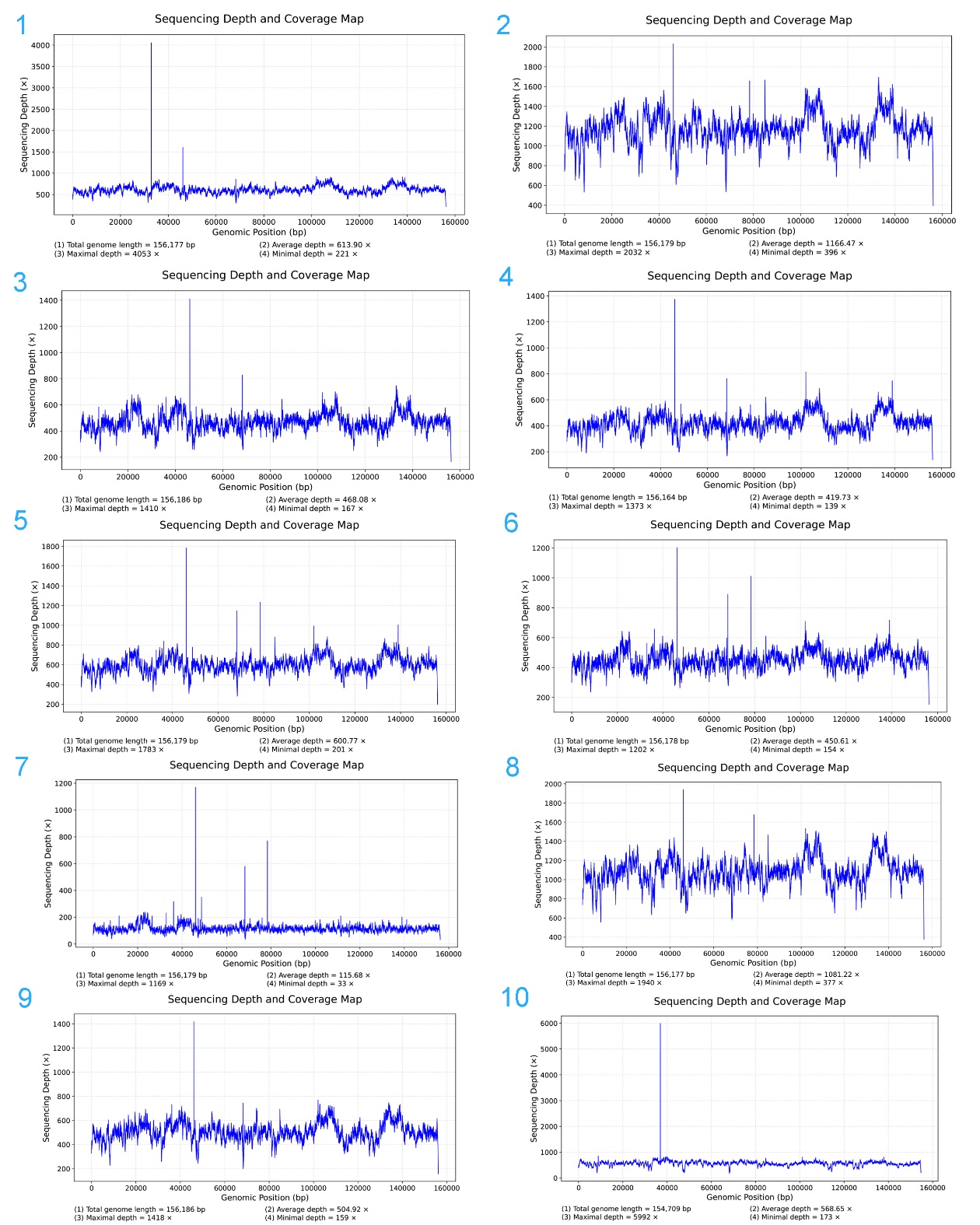


Fig S1. Displays the coverage depth of the chloroplast genomes of *Tamarix hispida* and *Myricaria bracteata*. Accessions GMbc, GMgg, GMhj, GMlt, GMwpe, GMxh, GMyl, GMyph, and GMzp represent *T. hispida* (1-9), while *M. bracteata* is represented by accession number 10.


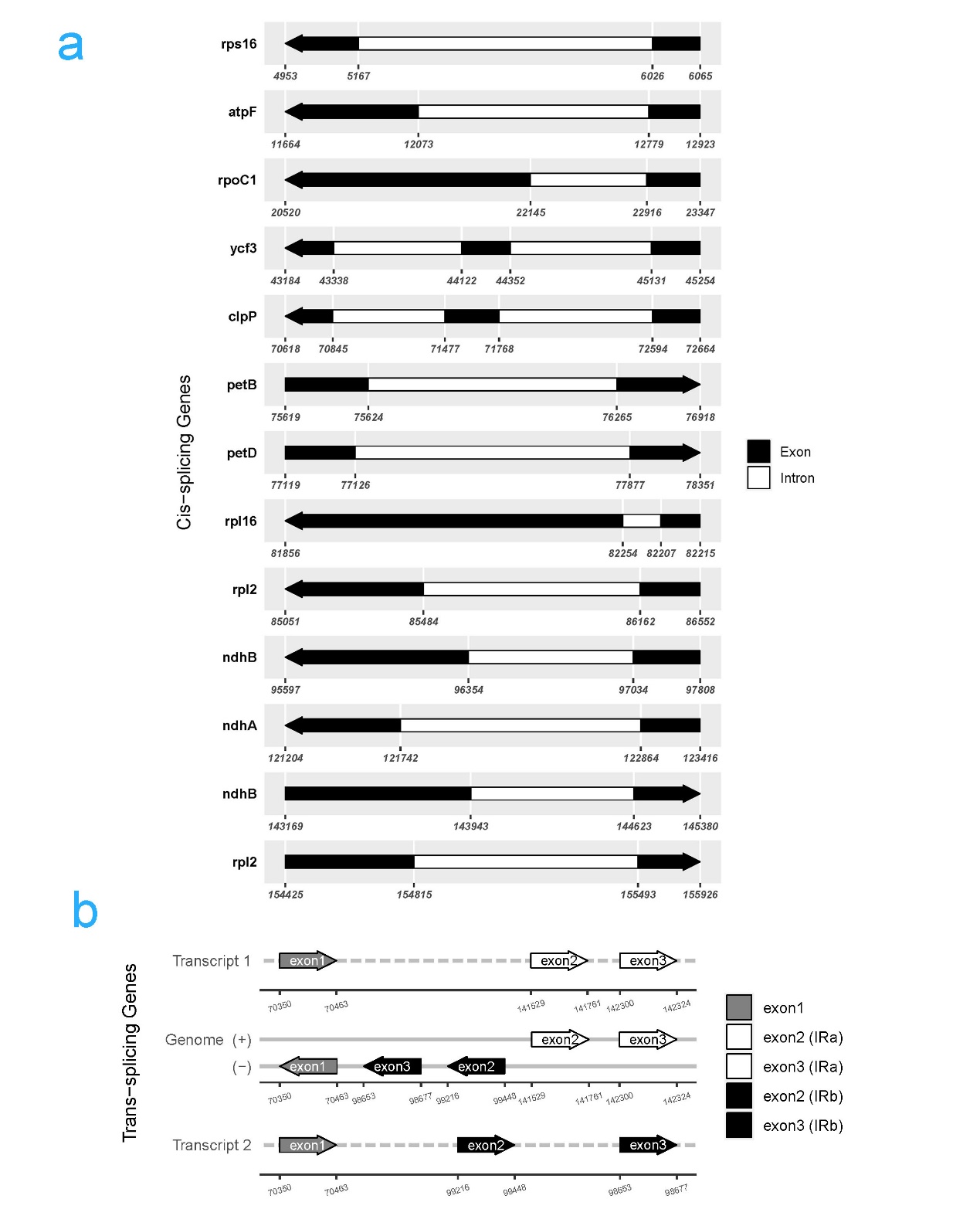


Fig S2. The schematic map illustrates the cis-splicing genes and the trans-splicing gene within the chloroplast genome of *Tamarix hispida* (GMbc).


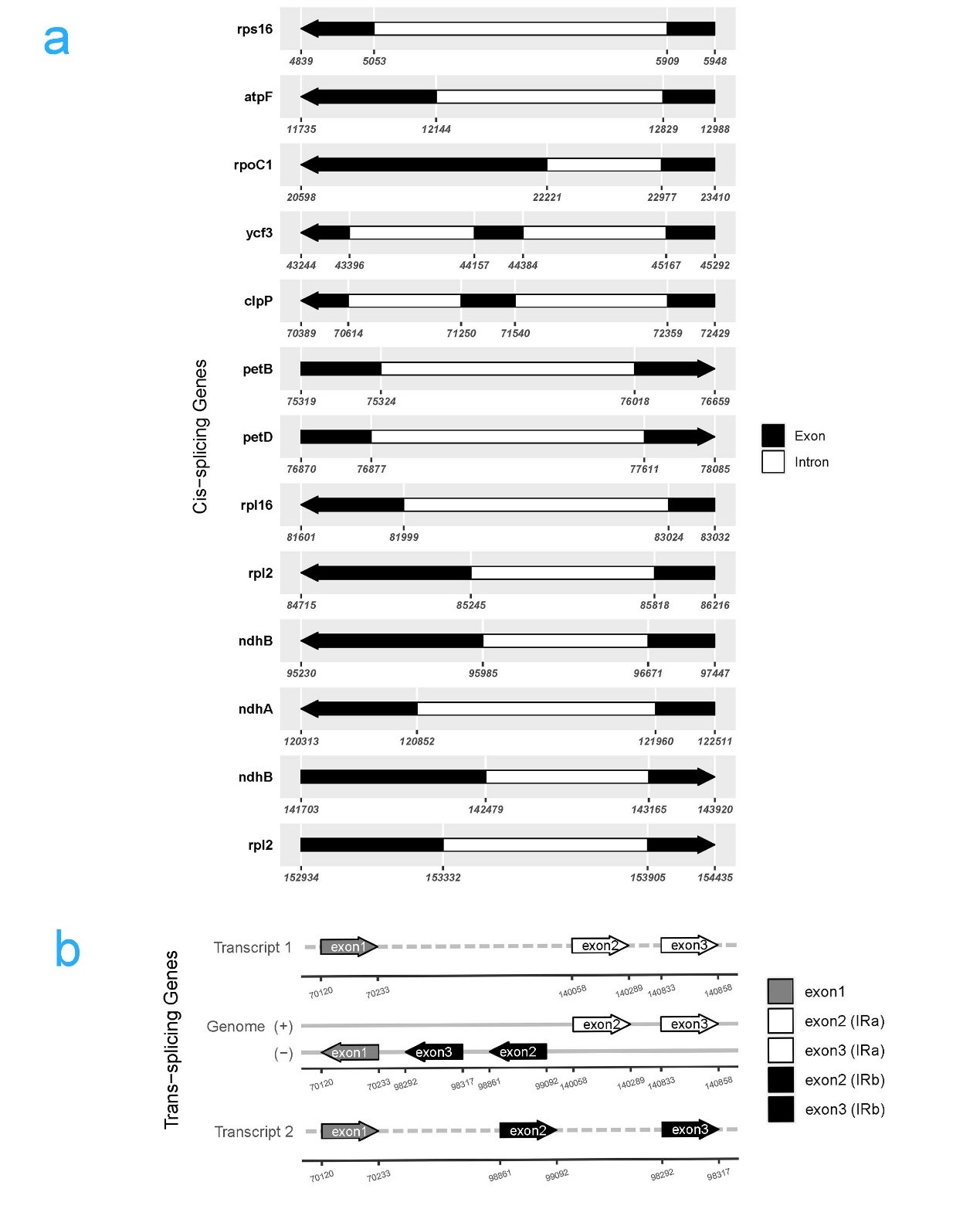


Fig S3. The schematic map illustrates the cis-splicing genes and the trans-splicing gene within the chloroplast genome of *Myricaria bracteata*.
